# Controlling Photocatalytic Methane Conversion Pathways: Challenges and Future Directions

**DOI:** 10.1021/acscentsci.6c00119

**Published:** 2026-04-09

**Authors:** Yingying Fan, Xiaoyan Jin, Zhiqing Guo, Yuheng Jiang, Siyang Li, Xiaoyu Fan, Jinghao Wang, Bastian Mei, Graham Hutchings, Zhiyong Tang

**Affiliations:** † Guangdong Engineering Technology Research Center for Sensing Materials & Devices, Guangzhou Key Laboratory of Sensing Materials & Devices, Center for Advanced Analytical Science, School of Chemistry and Chemical Engineering, Guangzhou University, Guangzhou 510006, P.R. China; ‡ Chinese Academy of Science (CAS) Key Laboratory of Nanosystem and Hierarchy Fabrication, CAS Center for Excellence in Nanoscience, National Center for Nanoscience and Technology, Beijing 100190, P. R. China; § Laboratory of Industrial Chemistry, Ruhr University Bochum, 44780 Bochum, Germany; ∥ University of Chinese Academy of Sciences Beijing, Beijing 100049, P. R. China; ⊥ Beijing Key Laboratory for Optical Materials and Photonic Devices, Department of Chemistry, Capital Normal University Beijing, Beijing 100048, P. R. China; # Max Planck-Cardiff Centre on the Fundamentals of Heterogeneous Catalysis FUNCAT, Cardiff Catalysis Institute, School of Chemistry, Cardiff University, Translational Research Hub, Maindy Road, Cardiff CF24 4HQ, U.K.

## Abstract

Photocatalytic methane conversion offers a sustainable route to transform the most inert C_1_ molecule into valuable oxygenates and hydrocarbons under ambient conditions. Recent progress has been made in the selective formation of methanol, ethanol, acetic acid, and C_2_ hydrocarbons, with notable efficiency. However, limited product diversity and an incomplete mechanistic understanding remain major barriers to further progress. This outlook deconstructs photocatalytic methane conversion into three elementary steps: formation of reactive species, coupling of reactive species, and transformation of intermediate products. This stepwise perspective enables a clearer identification of the factors governing individual reaction pathways and overall selectivity. By adopting a pathway-centric framework, the outlook integrates disparate observations from the literature into a unified mechanistic picture, elucidating how control over reactive-species generation, coupling modes, and intermediate evolution dictates reaction outcomes. From this analysis, general design principles and recurring control motifs are distilled, providing practical guidelines for the rational design of photocatalysts and reaction architectures aimed at more efficient and selective methane utilization.

## Graphical abstract

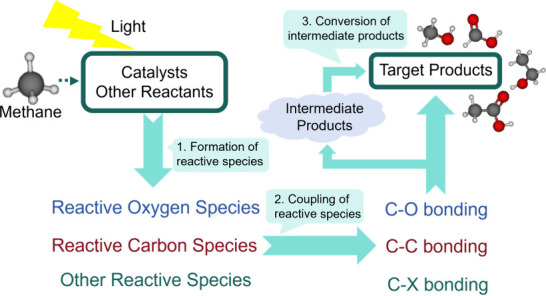

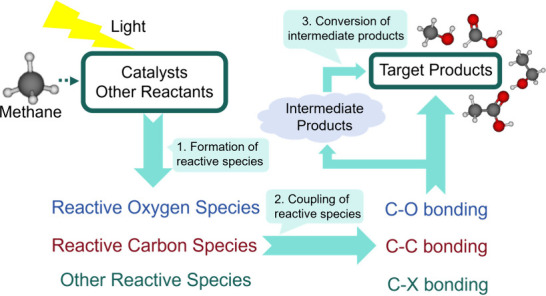

## 1 Introduction

The urgent need to reduce greenhouse gas emissions has drawn increasing attention to methane (CH_4_) utilization. Despite its atmospheric concentration being lower than that of CO_2_, CH_4_ is 28–34 times more potent as a greenhouse gas.^1–3^ At the same time, CH_4_ is the main component of natural gas, which is available in large reserves and serves as an important C_1_ feedstock for the chemical industry.^4–6^ For dilute CH_4_ emissions, such as those resulting from coal mines, direct oxidation to CO_2_ can already provide a practical route for climate mitigation. In contrast, for CH_4_-rich natural gas streams, routine flaring remains widespread, resulting in avoidable emissions and the loss of an otherwise valuable carbon resource. A nontrivial fraction of CH_4_ is wasted in this manner worldwide. Therefore, the direct conversion of CH_4_ into liquid, value-added products represent a compelling strategy to simultaneously reduce greenhouse gas emissions and improve resource utilization.

CH_4_ activation, however, remains intrinsically challenging and is often regarded as a “holy grail” in catalysis.^7–9^ Its high molecular symmetry and strong C–H bonds (bond dissociation energy ∼435 kJ·mol^–1^) demand substantial energy input. As a result, conventional industrial processes proceed through multistep, energy-intensive routes such as steam methane reforming (700–1100 °C) followed by Fischer–Tropsch synthesis (200–350 °C).^10,11^ These limitations have motivated the search for alternative activation strategies under milder conditions.

Among emerging strategies, photocatalytic CH_4_ conversion stands out for its ability to activate CH_4_ directly under mild conditions (<100 °C) using green oxidants or solvents (H_2_O or O_2_), offering superior selectivity and sustainability.^12–15^ Remarkable progress has been made in the selective formation of valuable C_1_ oxygenates via partial oxidation of CH_4_ (POM) and in the production of C_2+_ hydrocarbons through oxidative and nonoxidative coupling of CH_4_ (OCM/NOCM). For instance, in the context of gas–liquid–solid photocatalytic systems, studies have been conducted on benchmark C_1_ oxygenates, yielding productivities of 5 mmol·g^–1^·h^–1^ with over 80% selectivity for methanol (CH_3_OH).^16^ In gas–solid systems,^17^ ethane (C_2_H_6_) yields of up to 9 mmol·g^–1^·h^–1^ with over 90% selectivity have been demonstrated.^18^ Moreover, reports on C_2_ oxygenates such as ethanol (C_2_H_5_OH)^19,20^ and acetic acid (CH_3_COOH)^21,22^ are rapidly emerging.

However, performance metrics based solely on mass-normalized rates provide an incomplete picture. Apparent activities are often obtained using minute catalyst quantities under highly optimized laboratory conditions, and photon-to-chemical conversion efficiencies, such as apparent quantum yields (AQYs), remain modest in most systems.^23^ Moreover, product diversity is still narrow and mechanistic understanding fragmented, limiting meaningful comparisons across studies and obscuring scalability considerations. Existing reviews typically classify photocatalytic CH_4_ conversion by reaction type (POM, OCM, or NOCM), which can mask the shared elementary steps that ultimately govern reactivity and selectivity.^24,25^ Addressing these limitations requires a pathway-level understanding that connects light absorption, reactive-species generation, coupling chemistry, and intermediate transformation, thereby highlighting where fundamental knowledge gaps remain and where targeted advances are most urgently needed.

Addressing these limitations requires a pathway-level understanding that connects light absorption, reactive-species generation, coupling chemistry, and intermediate transformation, thereby highlighting where fundamental knowledge gaps remain and where targeted advances are most urgently needed.

This outlook deconstructs the overall CH_4_ conversion process into three key steps: this includes (1) the formation of reactive species, (2) the coupling of reactive species, and (3) the transformation of intermediate products. We analyze how strategic control over each of these elementary steps governs product distribution from a mechanistic perspective. Finally, the discussion focuses on current challenges and emerging research opportunities. The overarching objective of this pathway-centric framework is to enhance mechanistic comprehension, thereby facilitating the integration of diverse CH_4_ conversion systems and guiding the rational design of photocatalysts and reaction systems for efficient and selective CH_4_ utilization.

## 2 The Key Steps of Photocatalytic Methane Conversion

During photocatalytic CH_4_ conversion, the photocatalyst absorbs photons to generate electron–hole pairs. These photogenerated charge carriers subsequently react with CH_4_ and other reactants, initiating a series of reactions that produce value-added chemicals.^26^ This outlook primarily focuses on the interactions between CH_4_, coreactants, and charge carriers, as well as the subsequent chemical transformations, since these steps exert the greatest influence on product formation. The overall process can be conceptually divided into three key steps: (1) formation of reactive species, (2) coupling of reactive species, and an optional step involving (3) the conversion of intermediate products (Figure 1).

**Figure 1:**
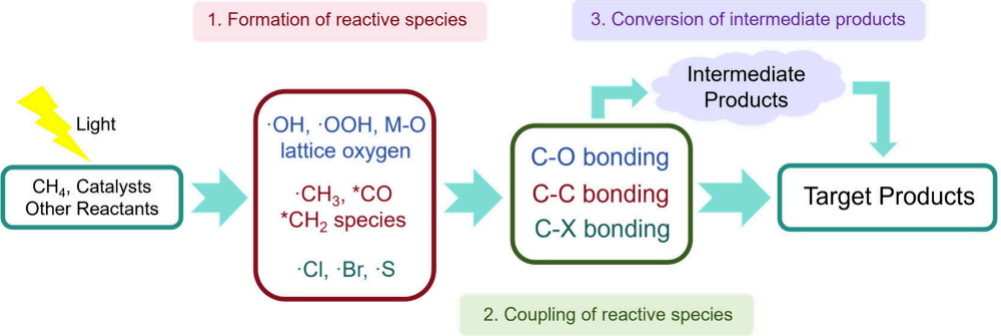
Three key steps of the photocatalytic CH_4_ conversion.

In the first step, stable reactant molecules (e.g., CH_4_, O_2_, H_2_O) are activated by the combined action of the photocatalyst and light, resulting in the formation of reactive species. A categorization of these reactive species, based on their chemical composition, is possible into three main types: reactive oxygen species, reactive carbon species, and other reactive species. Reactive oxygen species mainly include free radicals (e.g., ·OH, ·OOH), activated lattice oxygen (O_L_^–^), and metal–oxygen species (M–O), which can activate CH_4_ or participate directly in product formation. Reactive carbon species, such as ·CH_3_, CH_2_ species and *CO (*adsorbed species), are generated through CH_4_ activation and subsequently engage in coupling reactions to yield various intermediates and final products. Moreover, recent studies have revealed that the introduction of halide ions or sulfur-based organic catalysts can generate ·X^27^ or ·S^28^ radicals respectively, thereby providing new pathways and enabling the formation of novel products.

In the subsequent step, active species undergo bond-forming reactions to generate either the final product or intermediate species. Characteristic bond formation types include C–O bonds, C–C bonds, and C-X bonds as shown in Table 1. In this process, a competitive reaction between C–C and C–O coupling typically occurs, which decisively influences the selectivity of the pathway. In general, reaction conditions play a critical role in determining the competition pathways. Parameters such as O_2_ concentration, the presence of coreactants (e.g., CO or CO_2_), and the availability of surface oxygen species strongly influence the relative concentrations of oxidative species and carbon radicals. High oxidant availability generally favors C–O coupling and the formation of oxygenates, whereas lower oxygen activity or higher concentrations of carbon-containing coreactants increase the probability of C–C coupling toward multicarbon products.

**Table 1:** Representative C–O, C–C, and C–X Bond-Forming Reactions^a^

Coupling Reaction	Reactants	Products	ref
C–O coupling	·CH_3_ + ·OH	CH_3_OH	^34^
	·CH_3_ + ·OOH	CH_3_OOH	^37^
	·CH_3_ + O_2_	CH_3_OO·	^29^
	CH_4_ + O_L_^–^	*OCH_3_	^17^
	·CH_2_OH + ·OH	HOCH_2_OH	^36^
C–C coupling	·CH_3_ + ·CH_3_	C_2_H_6_	^31^
	·CH_3_ + *CO	*COCH_3_	^30^
	·CH_3_ + ·CH_2_OH	CH_3_CH_2_OH	^35^
C–X coupling	·CH_3_ + ·Cl	CH_3_Cl	^33^
	·CH_3_ + ·Br	CH_3_Br	^32^

a Asterisks (*) refer to adsorbed species and O_L_^–^ refers to the activated lattice oxygen.^17,29–37^

Follow-up reactions involving the conversion of intermediate products have been observed to occur exclusively within specific catalytic systems. For instance, C_2_H_6_ intermediates undergo dehydrogenation to produce ethylene (C_2_H_4_).^38^ Methyl hydroperoxide (CH_3_OOH) often serves as an intermediate that can be further transformed into CH_3_OH^39^ or formaldehyde (HCHO).^40^ Similarly, CH_3_OH and HCHO may undergo subsequent oxidation to form HCHO^41^ or formic acid (HCOOH),^42^ respectively. Moreover, CH_3_OH has been observed to participate in the reaction to C_2_H_5_OH.^19^

These three steps collectively define the overall reaction pathway. Among them, the first step is typically considered to be the rate-limiting step, as the formation of reactive species directly determines the overall reaction rate. In the second step, the competitive coupling of different reactive species predominantly governs reaction selectivity. The third step involves subsequent transformations of intermediate products, which ultimately dictate the yield and selectivity of the final products. These three elementary steps provide a mechanistic framework that also informs catalyst design principles. The formation of reactive species is largely governed by the photocatalyst’s light absorption properties, band structure, and charge separation efficiency, which determine the generation of reactive oxygen and carbon species. The coupling of reactive intermediates depends strongly on the nature and distribution of surface active sites, as well as the adsorption strength of key intermediates. Finally, the transformation of intermediate products is influenced by the spatial arrangement of catalytic sites and the local reaction environment. Establishing clear links between these mechanistic steps and catalyst properties provides a useful guideline for the rational design of photocatalytic systems for selective CH_4_ conversion.

## 3 Reactive Species Formation

The reaction of photogenerated charge carriers with reactants results in the generation of various reactive species, including reactive oxygen species (e.g., ·OH, ·OOH, lattice oxygen, and M–O), reactive carbon species (e.g., ·CH_3_, CH_2_ species and *CO), and other reactive species (e.g., ·X and ·S). The types and concentrations of these reactive species have been shown to have a significant impact on the performance of photocatalytic CH_4_ conversion. Their interactions with catalyst surfaces also shape the reaction pathways via the d-band center theory, as the position of the catalyst’s d-band center determines the bonding strength between the surface and reactant molecules, thereby regulating their adsorption and desorption.^43^ In this section, the formation and regulation of these reactive species, and their effects on the overall CH_4_ conversion process, will be discussed.

### 3.1 Reactive Oxygen Species

Reactive oxygen species are mainly free radicals, among which ·OH and ·OOH are the most common. In addition, lattice oxygen and metal–oxygen (M–O) species can also form on the catalyst surface.

#### 3.1.1 Free Radicals of ·OOH and ·OH

In photocatalytic CH_4_ conversion systems, *OOH can be generated through O_2_ reduction (Table 2, eq 1).^26^ In this pathway, the desorption process affects the selectivity of radicals, in which *OOH either decomposes into ·OH (Table 2, eq 2) or simply desorbs to form ·OOH (Table 2, eq 3). It is worth noting that ·O_2_^–^ and ·OOH are related reactive oxygen species that exist in a protonation–deprotonation equilibrium. When the pH is below 4.8, ·O_2_^–^ is protonated to form ·OOH. These two species can be distinguished by electron spin resonance (ESR) spectroscopy.^44^ Regulating the electronic and geometric structures of cocatalysts has proven to be an effective strategy for tuning this selectivity. Jiang et al. demonstrated that the size of supported metal species in Au/In_2_O_3_ systems dictated the preference for ·OOH or ·OH generation. On single-atom Au_1_ sites, O_2_ adopted an end-on adsorption configuration, which favored ·OOH formation, while on Au nanoparticles, side-on O_2_ adsorption facilitated ·OH generation (Figure 2a), as the low-temperature Fourier-transform infrared (FTIR) spectra and density functional theory (DFT) results showed. Consequently, HCHO was the dominant product over Au_1_/In_2_O_3_, whereas CH_3_OH prevailed over Au_NPs_/In_2_O_3_.^40^

**Table 2:** Redox Potentials (V vs. NHE, pH = 0) of Common Reactions in Photocatalytic CH_4_ Conversion^44–46^

Reaction Equation	Reduction Potential (V vs NHE, pH = 0)
1 O2+e−+H+→OOH*	–0.05
2 OOH*+H++e−→2·OH	1.90
3 OOH*→·OOH	–
4 $H_{2}O+h^{+}\to \cdot \mathrm{OH}+H^{+}$	2.38
5 ${\mathrm{CH}}_{4}+h^{+}\to \cdot {\mathrm{CH}}_{3}+H^{+}$	2.06
6 $O_{2}+2e^{-}+2H^{+}\to H_{2}O_{2}$	0.70
7 $2H_{2}O+2h^{+}\to H_{2}O_{2}+2H^{+}$	1.76
8 $H_{2}O_{2}+e^{-}\to \cdot \mathrm{OH}+{\mathrm{OH}}^{-}\;\left(\mathrm{major}\right)$	1.14
9 $H_{2}O_{2}\to 2\cdot \mathrm{OH}\;\left(\mathrm{minor}\right)$	–

**Figure 2:**
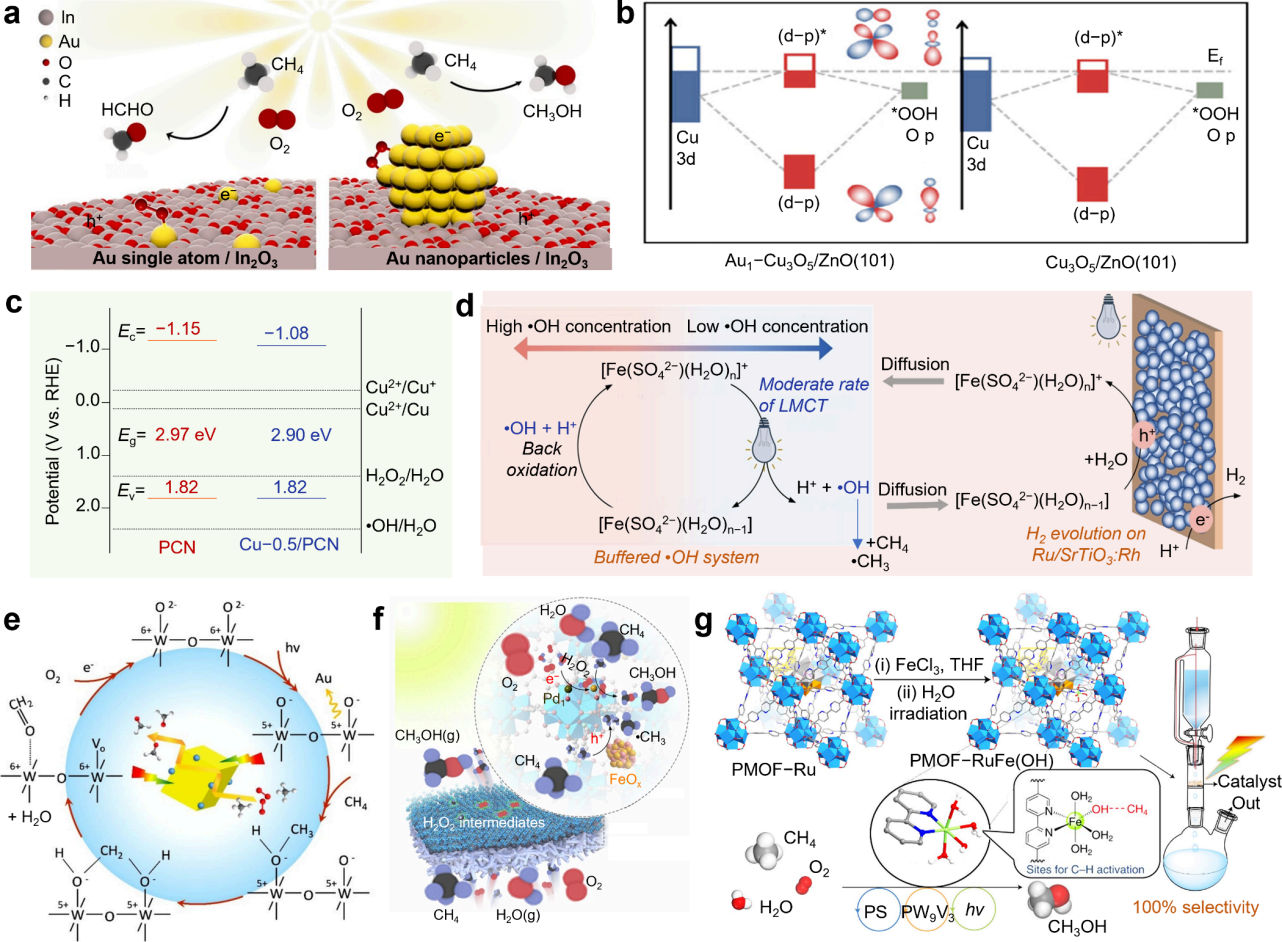
**The formation of reactive oxygen species.** (**a**) Proposed mechanism of photocatalytic conversion of CH_4_ to HCHO or CH_3_OH on Au_1_/In_2_O_3_ or Au_NPs_/In_2_O_3_, respectively.^40^ Adapted from ref (^40^). Copyright 2023 American Chemical Society. (**b**) Illustration of the orbital interactions between Cu centers and adsorbed *OOH on CuO_*x*_/ZnO and Au_1_–CuO_*x*_/ZnO, respectively.^48^ Adapted from ref (^48^). Copyright 2025 Wiley-VCH. (**c**) Band structure alignments of PCN and Cu-0.5/PCN.^19^ Adapted from ref (^19^). Copyright 2019 Zhou, Y. (**d**) Schematic diagram of a light-driven ·OH buffer that couples with Ru/SrTiO_3_:Rh for photocatalytic nonoxidative coupling of CH_4_.^55^ Adapted from ref (^55^). Copyright 2025 Wiley-VCH. (**e**) Proposed reaction mechanism for HCHO generation over cubic WO_3_.^59^ Adapted from ref (^59^). Copyright 2020 Elsevier. (**f**) Schematic illustration for photocatalytic tandem oxidation of CH_4_ into CH_3_OH over UiO-66–NH_2_ coloaded with Pd and Fe single atoms.^60^ Adapted from ref (^60^). Copyright 2025 Wang, Y. (**g**) View of the structure of PMOF-RuFe­(OH) incorporating monoiron hydroxyl, [Ru^II^(bpy)_2_(bpydc)] and [PW_9_V_3_O_40_]^6–^ species within the pore of UiO-67, view of PMOF-RuFe­(OH) for selective photoactivation of the C–H bond, view of the continuous-flow apparatus for photocatalytic conversion. Labeling scheme: C, gray; O, red; H, white; N, blue; Fe, green; V, orange; W, dark gray; P, purple; {Zr_6_} cluster, blue polyhedral; [Ru^II^(bpy)_2_(bpydc)] (PS), gold.^61^ Adapted from ref (^61^). Copyright 2022, An, B.

Building on this concept, several studies explored ZnO-based systems. Gong et al. reported that the single-atom Ru oxide (Ru_1_O_*x*_) species with a five-coordinate structure on ZnO preferentially reduced O_2_ to ·OOH, while for isolated Ru_1_/ZnO sites exhibited no notable selectivity. The Ru_1_O_*x*_ species activated O_2_ via end-on adsorption and possessed a high dissociation energy barrier for *OOH, achieving over 95% selectivity for alkyl hydroperoxide formation from C_1_–C_4_ alkanes.^47^ Si et al. further investigated the electronic factors influencing O–O bond activation, chiming with the d-band center theory mentioned above. Their computational analysis revealed that loading Au single atoms on CuO_*x*_/ZnO shifted the Cu d-band center upward (Figure 2b), enhancing *OOH adsorption through d–p orbital hybridization. Increased electron occupancy in antibonding orbitals weakened the O–O bond, promoting its cleavage to ·OH. As a result, the CH_3_OH yield of Au_1_–CuO_*x*_/ZnO was three times higher than that of CuO_*x*_/ZnO.^48^ Similarly, Zhou et al. found that over Au/ZnO, the {111} facet of Au, characterized by low surface free energy and high oxygen adsorption energy, predominantly favored ·OOH formation, whereas the {100} facet favored ·OH production.^49^ Furthermore, Pd–Au/ZnO exhibited a higher O_2_ adsorption energy and lower *OOH dissociation energy compared to Au/ZnO, enabling highly selective ·OH formation from *OOH.^26^

Besides from O_2_, ·OH radicals can also be generated from H_2_O (Table 2, eq 4). Water can be directly oxidized by photogenerated holes with the valence band potential of the photocatalyst being quite critical for this reaction (·OH/H_2_O = 2.38 V vs NHE). Optimizing the band structure provides an effective strategy to enhance ·OH generation. For instance, Fan et al. synthesized 4.5 nm quantum-sized BiVO_4_, where the quantum confinement effect widened the bandgap and shifted the valence band to a more positive potential (2.6 V vs NHE) compared to submicron-sized BiVO_4_ with a size of 454.3 nm (2.5 V vs NHE). This shift promoted H_2_O oxidation to ·OH by photogenerated holes.^36^ In theory, a more positive valence band position enhances the oxidation ability and thus facilitates higher ·OH production. However, competitive CH_4_ oxidation by holes must also be considered (CH_4_ + h^+^ → ·CH_3_ + H^+^, ·CH_3_/CH_4_ = 2.06 V vs NHE, Table 2, eq 5). To address this, Jiang et al. employed cubic In_2_O_3_ with a suitable valence band position (∼2.08 V vs NHE), which suppressed H_2_O oxidation to ·OH and directed hole oxidation exclusively toward CH_4_, selectively producing ·CH_3_ radicals.^40^ As was outlined above, by means of adjusting the generation of ·OH and ·OOH from O_2_, the selective oxidation of CH_4_ to CH_3_OH or HCHO can be achieved.

Another pathway for ·OH generation involves H_2_O_2_ as an intermediate, which can either be externally supplied or produced *in situ* through the 2-electron oxygen reduction reaction (ORR, see Table 2, eq 6). In addition, photogenerated holes can oxidize H_2_O to form H_2_O_2_ (Table 2, eq 7).^19^ The subsequent decomposition of H_2_O_2_ proceeds mainly through Fenton or Fenton-like pathways, in which H_2_O_2_ is heterolytically cleaved to generate ·OH and OH^–^ (Table 2, eq 8), whereas direct homolytic cleavage yielding two ·OH radicals is much less prevalent (Table 2, eq 9). Consequently, both the selective formation of H_2_O_2_ and its controlled *in situ* decomposition are critical for regulating ·OH availability.^50,51^ Polymeric carbon nitride (PCN) provides a representative platform for this strategy. Owing to its valence band potential of 1.82 V versus NHE (Figure 2c), PCN is thermodynamically capable of oxidizing H_2_O to H_2_O_2_ but not to ·OH directly. The introduction of mixed valence metal species further enables efficient Fenton like conversion of H_2_O_2_ into ·OH. For example, Zhou et al. prepared Cu-modified PCN (Cu–PCN), which contains Cu^0^/Cu^+^/Cu^2+^ redox pairs (Figure 2c). In this system, holes photogenerated by bandgap excitation of PCN oxidized H_2_O to produce H_2_O_2_, which was subsequently reduced by Cu^0^/Cu^+^ species to generate ·OH according to Cu^0^/Cu^+^ + H_2_O_2_ → Cu^+^/Cu^2+^ + ·OH + OH^–^. The resulting higher valence Cu species are then reduced back by photogenerated electrons, closing the redox cycle. This regulated ·OH generation led to a 5-fold increase in alcohol productivity compared with pristine PCN.^19^ A related but distinct strategy was reported by Wang et al., who reversed the redox sequence by first using high valence metal centers to capture photogenerated electrons and then employing the reduced metal species to activate H_2_O_2_. By decorating PCN with single-atom W, they exploited the reversible W^5+^/W^6+^ redox couple. Here, photogenerated holes oxidized H_2_O to form H_2_O_2_, while photoelectrons are trapped in W^6+^ leading to W^5+^, which subsequently reduced H_2_O_2_ to ·OH. The stepwise electron transfer pathway was found to be effective in avoiding excessive accumulation of H_2_O_2_. In addition, it enabled finely controlled ·OH generation, thereby effectively suppressing overoxidation and achieving 100% selectivity toward liquid oxygenates.^52^

The primary challenge in enhancing the ·OH yield through external introduction of H_2_O_2_ is the suppression of its nonproductive decomposition to O_2_. In this context, Fenton or Fenton-like reactions provide an effective strategy by selectively converting H_2_O_2_ into ·OH rather than molecular oxygen. For example, Xie et al. developed a FeO_*x*_/TiO_2_ catalyst that promoted H_2_O_2_ cleavage through the Fe^3+^/Fe^2+^ redox cycle, increasing CH_3_OH selectivity from 36% (pure TiO_2_) to over 90%.^53^ Furthermore, Xing et al. synthesized a FeN_*x*_/C catalyst combining Fe–N_*x*_ sites, i.e., Fe atoms coordinated with adjacent N atoms, and graphene-encapsulated Fe/Fe_3_C nanoparticles. Integration of the two structures into a close-knit architecture resulted in a synergistic catalytic effect. They found that Fe–N_*x*_ was the active site for the Fenton-like reaction to generate ·OH. Coexisting Fe_3_C nanoparticles in close proximity lead to an enhancement of the electron density of Fe–N_*x*_ to promote O–O bond cleavage, resulting in efficient CH_4_ oxidation to HCOOH with 90% selectivity.^54^

The ·OH radical possesses strong oxidative power, particularly in activating the C–H bond of CH_4_. However, its high reactivity also makes it prone to cause overoxidation; thus, fine-tuning both the concentration and adsorption state of ·OH is crucial. For instance, Wang et al. employed a coordination strategy as a ·OH buffer. In this system, Fe^3+^ in [Fe­(SO_4_)­(H_2_O)_n_]^+^ oxidized H_2_O to generate ·OH radicals through ligand-to-metal charge transfer (LMCT), while generated Fe^2+^ in [Fe­(SO_4_)­(H_2_O)_n‑1_] consumed excessive ·OH. The oxidation of Fe^2+^ to Fe^3+^ was facilitated by the accumulated holes in the catalyst Ru/SrTiO_3_:Rh, thereby completing the redox cycle (Figure 2d). The introduction of SO_4_^2–^ effectively slowed the LMCT process and maintained the ·OH concentration at a low level, thus suppressing overoxidation, reducing CO_2_ selectivity from 11% to below 1%.^55^ Another factor that was found to influence the CH_4_ oxidation performance is the adsorption state and spatial localization of ·OH. Villa et al. demonstrated that, in WO_3_-based systems, free ·OH in the bulk of the solution and surface OH groups led to different product selectivity. With pure WO_3_, ·OH was primarily adsorbed on the catalyst surface, producing CH_3_OH as the main product. Using fluorine-modified WO_3_, an increase in the free ·OH concentration was observed, which correlated with a decrease in the CH_3_OH yield and enhanced C_2_H_6_ formation.^56^ Both surface and free ·OH could activate CH_4_ to form ·CH_3_ radicals, and surface ·OH favored localized reactions, directing CH_3_OH formation. In contrast, free ·CH_3_ radicals underwent more random collisions, and C_2_H_6_ became the predominant product due to the lower activation energy of C–C coupling. The reactivity of adsorbed ·OH can also be modulated through crystal facet engineering.^57,58^ Ma et al. found that the distances between adsorbed ·OH species varied across different WO_3_ facets via DFT computation, leading to distinct radical activity. On the {100} facet, the closely spaced W atoms resulted in shorter ·OH–·OH distance, promoting hydrogen-bond formation and consequently reducing radical reactivity. In contrast, the larger W–W separation on the {010} facet maintained highly reactive ·OH species, favoring CH_4_ oxidation.^57^

#### 3.1.2 Activated Lattice Oxygen

Lattice oxygen refers to oxygen atoms embedded within the crystalline framework of metal oxides. Upon activation by photogenerated holes, these atoms can form O_L_^–^ species capable of cleaving the C–H bond in CH_4_ to generate surface-bound *CH_3_ intermediates.^59^ *CH_3_ may simply desorbs from the surface to form free ·CH_3_ radicals or react with lattice oxygen directly to form the product, with the resulting oxygen vacancies typically replenished by O_2_ or H_2_O. This process follows a Mars–van Krevelen (MvK) mechanism. However, unlike conventional thermocatalytic systems, in photocatalysis the lattice oxygen is activated by photogenerated holes, and the resulting oxygen vacancies can be replenished by photoinduced oxygen species with high mobility. This light-driven oxygen activation and regeneration enable the reaction to proceed efficiently at room temperature. For instance, in the cubic-WO_3_ system, O_L_^–^ species attacked surface-adsorbed ·CH_3_, producing stepwise intermediates (−O_L_–CH_3_ and −O_L_–CH_2_–O_L_−). These intermediates are subsequently converted into HCHO, achieving nearly 100% selectivity in the presence of O_2_ (Figure 2e).^59,62^

Doping represents an effective strategy for modulating lattice oxygen activity. Zhang et al. tuned the metal–oxygen bond energy by introducing Si into TiO_2_, thereby suppressing lattice oxygen participation. In their Pd_1_/ST (single atom Pd-modified and Si-doped TiO_2_) system, Si facilitated the formation of SiO_4_ units which stabilized the lattice oxygen and weakened the bonding between CH_4_ and O_L_ site, resulting in a reduced CO_2_ yield compared with Pd_1_/TiO_2_.^63^ Sun et al. further modulated the M–O_L_ bond strength by doping Ce into an Au/ZnO system. Within the Zn–O_L_–Zn–O_L_–Ce framework, hybridization between Ce 4*f*/5*d* and O 2p orbitals, along with doping-induced tensile strain was observed by Raman spectroscopy, leading to a suppressed lattice oxygen activity in −Zn–O_L_–Ce while oxygen activity was enhanced for Zn–O_L_–Zn–. This asymmetric electron distribution facilitated *CH_3_ desorption, promoting ·CH_3_ transporting for C–C coupling.^64^ To confirm the lattice oxygen pathway, Li et al. employed Zn^16^O/^18^O_2_ isotopic labeling on a single-atom Ru_1_/ZnO photocatalyst. Using Time-of-Flight Secondary Ion Mass Spectrometry (TOS-SIMS), the ratio of ^18^O to ^16^O at ZnO surface in ^18^O_2_ atmosphere was found to increase after illumination, which confirmed that O_2_ diffusion from the gas phase into the ZnO bulk. Importantly, in this system Ru_1_ loading enhanced the single-electron activation of O_2_ to ·O_2_^–^ and enabled regeneration of lattice oxygen vacancies, thereby improving both catalytic activity and stability.^65^

#### 3.1.3 M–O Species

Under light irradiation, M–O bonds are typically formed from the interaction between oxidants (e.g., H_2_O_2_, O_2_ or H_2_O) and catalyst surface sites, mediated by photogenerated charge carriers, which subsequently react with CH_4_ on the catalyst surface to selectively generate oxygenates. For example, Hao et al. synthesized UiO-66–NH_2_ coloaded with Pd and Fe single atoms (Figure 2f). Photogenerated electrons accumulate on Pd sites reduced O_2_ to H_2_O_2_, while holes captured by adjacent Fe sites, enabled the formation of high-valent Fe^IV^=O species via reaction with H_2_O_2_, which was proved via situ attenuated total reflectance FTIR spectroscopy. This Fe^IV^=O species activated CH_4_ into ·CH_3_ and were reduced to Fe^III^–OH, thereby completing the redox cycle and suppressing nonselective oxidation pathways.^60^ M–O systems effectively suppress overoxidation by avoiding the free radical pathway. Feng et al. reported that for Ag-decorated TiO_2_ catalyst, Ti–O–Ti sites on the TiO_2_{001} surface could form Ti–OO–Ti superoxide species upon O_2_ adsorption under illumination, which has been revealed by operando FTIR spectroscopy. Solid-state ex-situ ^17^O NMR experiments proved the formation of Ti–OCH_3_HO–Ti intermediates from Ti-OO-Ti activated CH_4_, rather than the formation of ·CH_3_ and ·OH, which was yielded. Subsequently, Ti–OCH_3_HO–Ti released CH_3_OH to regenerate the Ti–O–Ti motif. Compared to {101} facets with less distorted Ti–O–Ti bond angles, which consequently possessed fewer active Ti–O–Ti sites, the enhanced stability of the intermediates on {001} facets resulted in a 3-fold increase in CH_3_OH production rate.^17^ Notably, the formation of M–O species can also occur during the catalyst synthesis process. An et al. developed a monoiron hydroxyl sites immobilized within a metal–organic framework, PMOF–RuFe­(OH) (Figure 2g), where monoiron hydroxyl sites were generated via photolytic hydrolysis of the PMOF–RuFe­(Cl) precursor. X-ray photoelectron spectroscopy analysis confirmed the presence of Fe–OH species, which formed Fe–OH···CH_4_ intermediates with CH_4_ through hydrogen bonding, followed by homolytic C–H bond cleavage to produce ·CH_3_ radicals. DFT calculations revealed that Fe–OH sites not only lowered the C–H bond activation barrier but also stabilized the resulting ·CH_3_ radicals, leading to highly selective CH_3_OH formation with 100% selectivity.^61^

### 3.2 Reactive Carbon-Containing Species

The most common reactive carbon species in photocatalytic CH_4_ conversion is the ·CH_3_ (as shown in Figure 3a), which forms via single hydrogen abstraction from CH_4_ and serves as the fundamental building block in the subsequent reaction pathways. The methylene species (*CH_2_ or −CH_2_−), generated through double hydrogen abstraction, is thermodynamically less stable and thus less frequently observed. Nevertheless, recent studies have identified *CH_2_ and *CH_2_OH as key intermediates in the C_2_ oxygenates formation. In addition, carbon-containing species in higher oxidation states, such as *CO, have also been detected in some systems. Undoubtedly, the generation and transformation of these carbon-centered intermediates play a decisive role in directing CH_4_ conversion.

**Figure 3:**
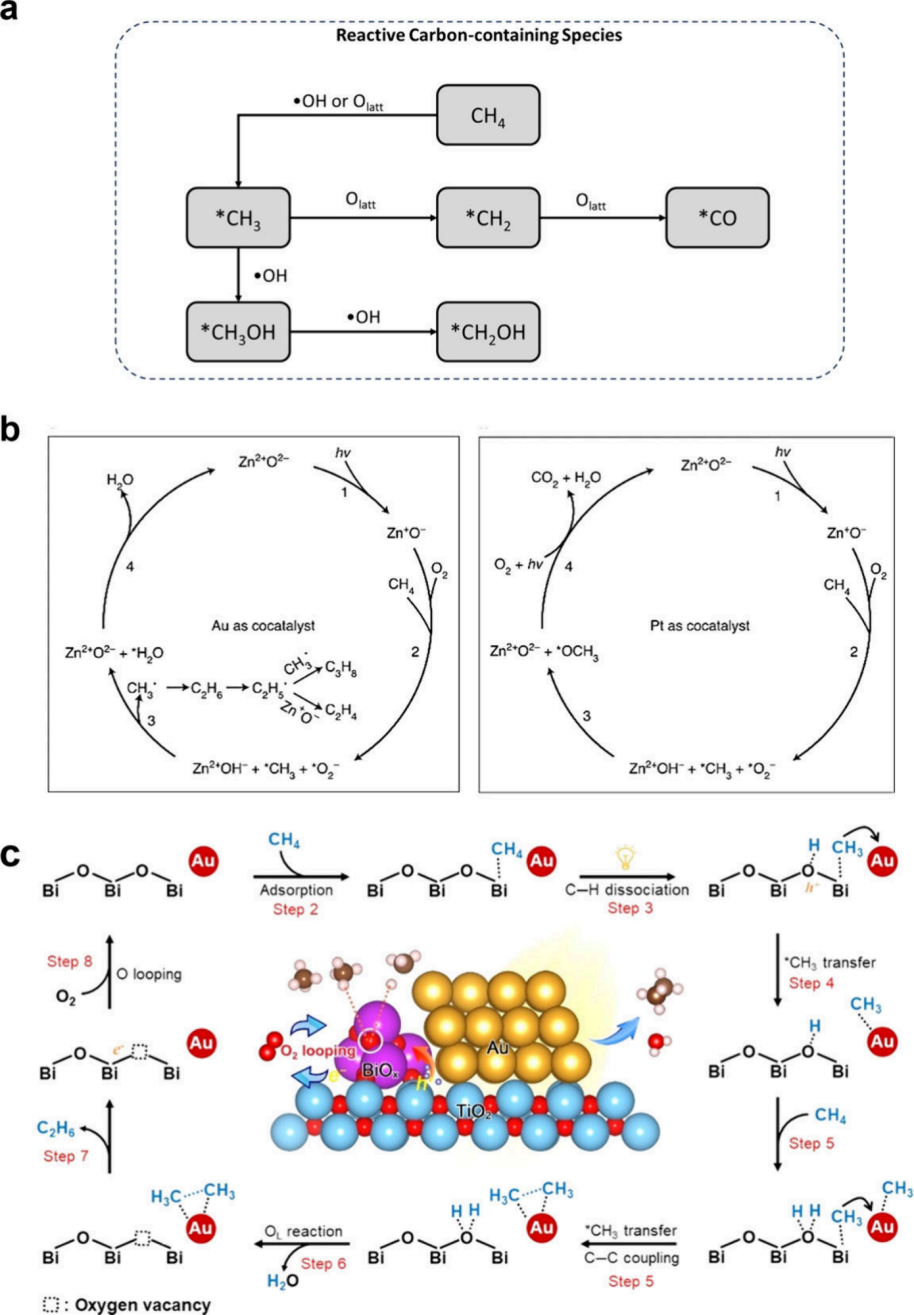
**The formation of reactive carbon species.** (**a**) Representative reactive carbon-containing species and their formation pathways. (**b**) Proposed reaction process on ZnO loaded with Au cocatalyst (left) and on ZnO loaded with Pt cocatalyst (right).^73^ This mechanism is based on DFT calculation, in which only the model of ZnO was used to simplify the calculation process. Adapted with permission from ref (^73^). Copyright 2025 Springer Nature. (**c**) Proposed reaction pathway for the light-driven OCM to C_2_H_6_ product over Au/BiO_*x*_-TiO_2_.^18^ Adapted with permission from ref (^18^). Copyright 2024 The American Association for the Advancement of Science.

#### 3.2.1 CH_3_ Species

The formation of ·CH_3_ requires activation of the strong C–H bond in CH_4_. Thermodynamically, hydrogen abstraction becomes feasible only when the reactive oxygen species provide sufficient driving force.^66,67^ Both the nature of the oxidizing species and the catalytic surface properties jointly determine the feasibility of ·CH_3_ formation.

Among the oxidizing species in photocatalytic CH_4_ activation, ·OH is one of the most powerful hydrogen abstractors, enabling rapid C–H cleavage through a highly exothermic process (ΔH = −60 kJ·mol^–1^) with a low activation energy of only 15 kJ·mol^–1^.^68^ However, the high reactivity of free radicals often leads to poor selectivity and significant overoxidation of ·CH_3_ intermediates. In contrast, lattice oxygen (O^–^) provides more controlled activation because its redox properties and adsorption behavior can be tuned through catalyst composition and defect engineering, although continuous oxygen replenishment is required to maintain structural stability. Metal–oxo (M–O) species offer even greater site-specific control over CH_4_ activation, but the long-term stability of these sites under photocatalytic conditions remains a challenge. Overall, ·OH-mediated activation favors high activity, whereas lattice oxygen and M–O pathways provide better selectivity control but require careful management of catalyst stability.^69^

This Outlook deconstructs the overall CH_4_ conversion process into three key steps: this includes (1) the formation of reactive species, (2) the coupling of reactive species, and (3) the transformation of intermediate products.

The fate of methyl radicals (·CH_3_) is largely determined by their partitioning between free radicals in the reaction phase and surface-bound intermediates, which depends on catalyst properties and reaction environment (e.g., gas–solid versus gas–liquid–solid systems). When ·CH_3_ desorbs into the gas or liquid phase, its high mobility favors radical recombination, typically producing C_2_H_6_, a pathway commonly observed in gas–solid systems with weak ·CH_3_ adsorption and limited oxidant availability. In contrast, surface-bound *CH_3_ tends to react with oxygen species on the catalyst surface, promoting C–O coupling and the formation of oxygenates such as CH_3_OH, HCHO, or CH_3_COOH, particularly in gas–liquid–solid systems where water and dissolved oxygen generate oxidative species. In addition, stabilized *CH_3_ may undergo dehydrogenation to *CH_2_, enabling further C–C coupling to form products such as C_2_H_4_ or C_2_ oxygenates. Under strongly oxidative conditions or long residence times, carbon intermediates can undergo deep oxidation to CO or CO_2_.^70,71^ Thus, the balance between radical desorption and surface confinement of CH_3_ intermediates is a key factor governing product selectivity in photocatalytic CH_4_ conversion.

The binding strength of absorbed *CH_3_ intermediates can be tuned through cocatalyst engineering and are often rationalized by electronic descriptors such as the cocatalyst d-band position, typically evaluated by DFT calculations.^72^ Taking the CH_4_ coupling as an example, overly strong adsorption prolongs surface residence and promotes sequential oxidation of *CH_3_, while moderate binding facilitates release or controlled steering of methyl species, increasing the probability of C–C coupling. This effect is exemplified in the Au/ZnO/TiO_2_ system for OCM.^73^ Compared with other metals, Au nanoparticles performed exceptionally well as electron sinks, facilitating the desorption of *CH_3_ species as ·CH_3_ radicals that subsequently couple to form C_2_H_6_ (Figure 3b). In contrast, stronger binding sites such as Pt stabilized ·CH_3_ its surface-mediated oxidation to CO_2_. Similarly, Zhai et al.^18^ constructed an Au/BiO_*x*_-TiO_2_ hybrid photocatalyst, in which BiO_*x*_ formed an oxygen-active surface layer on TiO_2_ and Au nanoparticles were positioned adjacent to the BiO_*x*_ clusters, enabling coordinated C–H activation and radical steering. In this composite photocatalyst, Au sites adjacent to lattice oxygen of the BiO_*x*_ cluster trapped ·CH_3_ while preventing overoxidation of newly formed ·CH_3_ radicals (Figure 3c). Comparatively, the ·OH-mediated oxidation pathway lacked the capacity for such fine control over the downstream reactivity of ·CH_3_. Taken together, these examples show that the same cocatalyst metal can exhibit different methyl adsorption–desorption behavior on different supports, thereby leading to distinct reaction pathways. Accordingly, designing the catalyst for CH_3_-formation pathway must be guided by the desired product network to maximize productivity and selectivity.

Beyond cocatalyst engineering, external physical factors such as the reaction temperature influence the surface residence time of ·CH_3_ radicals. To illustrate this point, slightly elevated temperatures can provide sufficient kinetic energy for adsorbed ·CH_3_ to escape the surface potential well, thereby facilitating efficient desorption into the gas phase and suppressing further undesired oxidation.^73–75^

#### 3.2.2 CH_2_ species

In photocatalytic CH_4_ conversion, species of the CH_2_–type primarily include *CH_2_ and *CH_2_OH. In gas–solid system, *CH_2_ has been identified as the key intermediate for C_2_H_4_ formation. It typically originates from *CH_3_ through stepwise dehydrogenation facilitated by cocatalysts with suitable dehydrogenation ability. For example, Liu et al.^76^ engineered synergistic Pd–Zn sites on defective WO_3_ nanosheets for photocatalytic nonoxidative CH_4_ coupling. The Pd sites promoted the dehydrogenation of *CH_3_ to *CH_2_, achieving a remarkable C_2_H_4_ selectivity of 75.3%. Owing to its d-band electronic structure, Pd provided an optimal balance between hydrogen adsorption and desorption, thereby serving as an efficient promoter for C_2_H_4_ formation. Beyond C_2_ pathways, CH_2_–like intermediates can also participate in carbene-type chemistry that drives higher-order homologation reactions. Zhang et al.^77^ reported that closely spaced Pt single atoms on black TiO_2_ could convert CH_4_ predominantly into propane (up to 1,440 μmol g^–1^ h^–1^ with 65% selectivity) under visible light, whereas isolated single-atom Pt sites showed much lower activity and no C–C growth. Time-resolved analysis of products revealed that C_2_H_4_ was predominantly formed at early stages, followed by constant accumulation of propane, suggesting a highly reactive C_1_ intermediate as the chain-growth intermediate. They further introduced C_2_H_4_ during photocatalysis and detected cyclopropane, which was interpreted as the presence of a methyl-carbene-like CH_2_ intermediate formed cooperatively on adjacent Pt centers (Figure 4a). This work points to the intriguing possibility of integrating carbene chemistry into photocatalytic CH_4_ conversion, thereby substantially expanding the accessible reaction space beyond conventional C_2_-coupling pathways.

**Figure 4:**
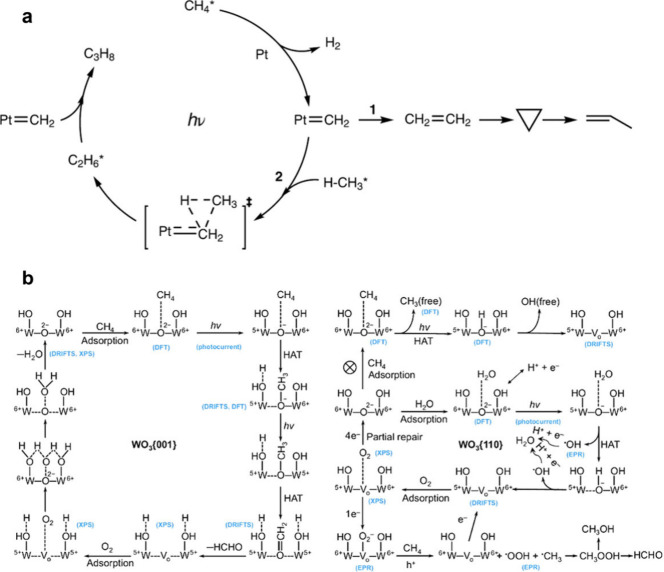
**The formation of CH**_**2**_ **species.** (**a**) The overall mechanism for propane formation over Pt-modified black TiO_2._ * indicates adsorption on Pt; *hv* indicates light irradiation; path 1 indicates carbene dimerization and path 2 shows carbene C–H insertion.^77^ Adapted with permission from ref 77. Copyright 2022 Springer Nature. (**b**) The schematic illustration of the proposed mechanism for photocatalytic oxidation of CH_4_ on WO_3_{001} and WO_3_{110}. V_o_ is the oxygen vacancy.^78^ Adapted with permission from ref (^78^). Copyright 2024 Springer Nature.

In liquid-phase systems, *CH_2_ is also reported as a crucial precursor to HCHO, representing a distinct reaction route from the conventional oxidation pathway where HCHO forms via CH_3_OOH/CH_3_OH intermediates. In this alternative mechanism, *CH_2_ is first generated by hydrogen abstraction from *CH_3_, followed by a MvK step, during which lattice oxygen inserts into *CH_2_ to form *HCHO. Compared with the radical oxidation pathway, this lattice oxygen–mediated mechanism offers inherently higher selectivity toward HCHO. To elucidate the factors governing these divergent pathways, Fan et al.^78^ studied the HCHO generation mechanism on the {001} and {110} facets of WO_3_ (Figure 4b). The low-surface-energy {001} facet, with higher oxidation potential and stronger CH_4_ adsorption, stabilized lattice oxygen and promotes direct MvK insertion of lattice O into *CH_2_, leading to high selective HCHO formation. In contrast, the high-surface-energy {110} facet, characterized by undercoordinated surface atoms and a lower oxygen-formation energy, more readily activated adsorbed H_2_O to generate ·OH radicals, which favored a stepwise, radical-mediated oxidation pathway.

Another important CH_2_–type species is *CH_2_OH, commonly identified as a key intermediate in selective C_2_H_5_OH formation. Hao et al.^79^ demonstrated that the electronic structure of the catalytic site is critical in determining the reaction pathway toward *CH_2_OH. By precisely tuning the phase composition of Zn–Fe complex oxides (Fe_2_O_3_/ZnO, ZnFe_2_O_4_/ZnO, Fe_3_O_4_/ZnO) to induce controlled lattice distortion, they synthesized Zn–O–Fe catalysts featuring low-, medium-, and high-spin Fe­(III) sites (denoted as LS, MS, and HS, respectively). Notably, Zn–O–Fe­(MS) selectively promoted *CH_2_OH formation from CH_3_OH, whereas the LS sites suppressed CH_3_OH dehydrogenation and the HS sites favored hydrogen abstraction from hydroxyl groups. DFT calculations attributed this behavior to the electronic configuration (*t*_*2*_ *g* ^4^ *e*_*g*_^1^) of Fe­(MS) centers, which provided an optimal density of unpaired electrons and an *e*_*g*_ orbital occupancy suitable for stabilizing the *CH_2_OH intermediate.

Overall, CH_2_–type species can evolve along multiple pathways, leading either to C–C coupling products such as C_2_H_4_ or to oxygenates such as HCHO and C_2_H_5_OH. The selective formation of *CH_2_ species is governed by the catalyst’s ability to stabilize the *CH_2_ intermediate and facilitate thermodynamically favorable hydrogen abstraction.

#### 3.2.3 CO Species

*CO is an important species in CO formation and *CO is also required for coupling with other carbon species to multicarbon products such as CH_3_COOH. However, the selectivity control is a challenge, because *CO can arise through stepwise oxidation of CH_4_ but overoxidation during the process is to be circumvented. A promising approach is to employ spatially separated tandem interfaces, in which distinct surfaces or interfaces are assigned to different elementary steps and connected through a confined architecture. For instance, Zheng et al.^80^ developed an asymmetric hollow multishell CeO_2_@PdO@FeO_*x*_ nanosphere photocatalyst, in which the PdO and FeO_*x*_ layers formed an outer catalytic interface while a CeO_2_ core coated with PdO formed an inner interface. In this composite material, CH_4_ was observed to be activated to *CH_3_ at the outer PdO/FeO_*x*_ interface, followed by further oxidation to *CO at the inner CeO_2_/PdO interface. The confined hollow structure enriched locally generated *CO and suppressed its premature desorption, thereby stabilizing this key intermediate. Similarly, a PdO/Pd–WO_3_ heterostructure was also shown to produce surface-bound *CO via the PdO lattice oxygen, while adjacent metallic Pd sites acted as adsorption site, further ensuring *CO availability.^14^

### 3.3 Other Reactive Species

In addition to reactive oxygen- and carbon-containing species also other radicals, including reactive halogen and sulfur species, have been reported to play significant roles in photocatalytic CH_4_ conversion. An et al. reported that sulfone-functionalized conjugated organic polymers could promote CH_4_ conversion through a radical-mediated pathway. In their sulfone-decorated conjugated organic polymers S-CTTP catalyst, light irradiation induced the homolytic cleavage of S=O bonds, generating ·O and ·S radicals. This allowed for CH_4_ activation by ·O, whereas the reduction of O_2_ to H_2_O_2_ was facilitated by ·S. Subsequent decomposition of H_2_O_2_ produced ·OH to further oxidized CH_4_ into CH_3_OH and HCOOH.^28^ Furthermore, in a halogen-based system, Wang et al. designed an oxygen-deficient BiOCl with H_2_O_2_ in the NaCl solution. The effective activation of CH_4_ to ·CH_3_ was achieved through the *in situ* generation of ·Cl from Cl^–^ ions. Moreover, the generation of H_2_O_2_ resulted in the formation of ·OH and ·OOH, which functioned as acceptors for ·CH_3_, thereby driving the selective formation of CH_3_OH.^27^

## 4 The Coupling of Active Species

Once reactive species are generated, their subsequent coupling reactions determine the pathway of CH_4_ conversion. Among these, two primary routes dominate: C–C coupling, which yields multicarbon hydrocarbons and oxygenates, and C–O coupling, which leads to selective oxidation products such as CH_3_OH and HCHO. Therefore, achieving precise control over the competition between C–C and C–O bond formation is crucial for directing the overall product selectivity. In practice, the relative rates of these channels depend on the relative abundance of carbon-centered versus oxygen-containing intermediates concentration.

Besides catalyst design, several experimentally tunable parameters play a crucial role in regulating the relative abundance of carbon-centered and oxygen-containing intermediates. In particular, oxidant concentration (e.g., O_2_ partial pressure and H_2_O content) strongly influences the balance between reactive oxygen species and ·CH_3_. Higher oxidant availability generally promotes the formation of oxidative species, favoring C–O coupling pathways, whereas lower oxygen activity increases the probability of radical recombination and C–C coupling. H_2_O also serves as a protective medium: sufficient H_2_O content facilitates the desorption and solvation of polar oxygenates such as CH_3_OH, rapidly removing them from catalytic sites and suppressing their further oxidation to CO_2_. Meanwhile, light intensity and cocatalyst loading determine the generation rate and surface residence time of reactive intermediates; excessive photon flux or high cocatalyst density can lead to the accumulation of surface-bound species, increasing the likelihood of deep oxidation. In addition, certain cocatalysts or additives (e.g., Cu^2+^ or NO_2_^–^)^81,82^ can modulate the concentration of reactive oxygen species, thereby influencing the competition between oxidation and coupling pathways. Therefore, careful tuning of these operational parameters is essential for controlling intermediate populations and optimizing selectivity in photocatalytic CH_4_ conversion.

### 4.1 C–C Coupling

After the formation of various reactive carbon species (e.g., ·CH_3_, *CH_2_, and *CO), their subsequent C–C coupling becomes the crucial step in generating multicarbon hydrocarbons and oxygenates. Among these routes, the radical self-coupling of ·CH_3_ to form C_2_H_6_ is the most common, occurring either in the reaction medium or at the catalyst surface from weakly adsorbed ·CH_3_. The electronic interaction between the catalyst and CH_3_ intermediates plays a crucial role in determining coupling efficiency. For example, Zhang et al. demonstrated that the formation of C_2_H_6_ in metal/ZnO photocatalysts could be controlled by tuning the adsorption strength of *CH_3_ on cocatalysts surface.^83,84^ In this system, CH_4_ was first activated on ZnO to form methyl species, which subsequently migrated to adjacent metal cocatalyst sites for coupling. The nature of *CH_3_-metal interaction governed the OCM products distribution: strong d-σ orbital hybridization, as observed in Au/ZnO, reduced the metal–C–H bond angle and steric hindrance, thereby facilitating efficient methyl coupling.

Coupling between ·CH_3_ and other carbon species can also yield oxygenates such as C_2_H_5_OH or CH_3_COOH. Because C–C coupling directly competes with further oxidation of carbon intermediates, suppressing overoxidation, especially the conversion of ·CH_3_ into C_1_ oxygenates or CO_2_, is essential for promoting C–C bond formation. Thus, the efficiency of C–C coupling must be understood together with the regulation of C–O coupling processes, which are discussed in the following section.

### 4.2 C–O Coupling

After reactive oxygen species (such as ·OH, ·OOH, lattice oxygen, and M–O) and reactive carbon species (such as ·CH_3_, CH_2_ species, and *CO) are generated, these species can undergo either hydrogen abstraction or C–O bond formation, and the specific mode of C–O coupling determines the reaction pathway and product distribution. This section therefore focuses on the mechanisms governing C–O bond formation.

·CH_3_ radicals formed from CH_4_ activation can couple with different oxygen-containing species, including O_2_, ·OOH, ·OH, and lattice oxygen. Typically, ·CH_3_ couples with O_2_ to form CH_3_OO·, which then converts into CH_3_OOH, or directly reacts with ·OOH to yield CH_3_OOH.^85^ When ·CH_3_ reacts with ·OH, CH_3_OH is formed. Distinct from free radical processes, lattice oxygen can abstract hydrogen from CH_4_ to produce surface-bound *OCH_3_, which subsequently desorbs as CH_3_OH (Figure 5a). Once CH_3_OH is formed, it can be reactivated to ·CH_2_OH, which couples with ·OH to generate HOCH_2_OH, a common precursor of HCHO.^86^ The regulation of this sequential oxidation process enables the modulation of selectivity between CH_3_OH and HCHO. For instance, Fan et al. demonstrated that for BiVO_4_ photocatalysts the reaction conditions were guiding product selectivity: continuous visible-light irradiation for 3 h yielded CH_3_OH with 92.8% selectivity, whereas irradiation with UV light for 7 h promoted further oxidation to HCHO with 86.7% selectivity (Figure 5b).^36^ Importantly, the generated ·CH_2_OH can also couple with ·CH_3_ to form C_2_H_5_OH, which will be discussed in Section 4.3.

**Figure 5:**
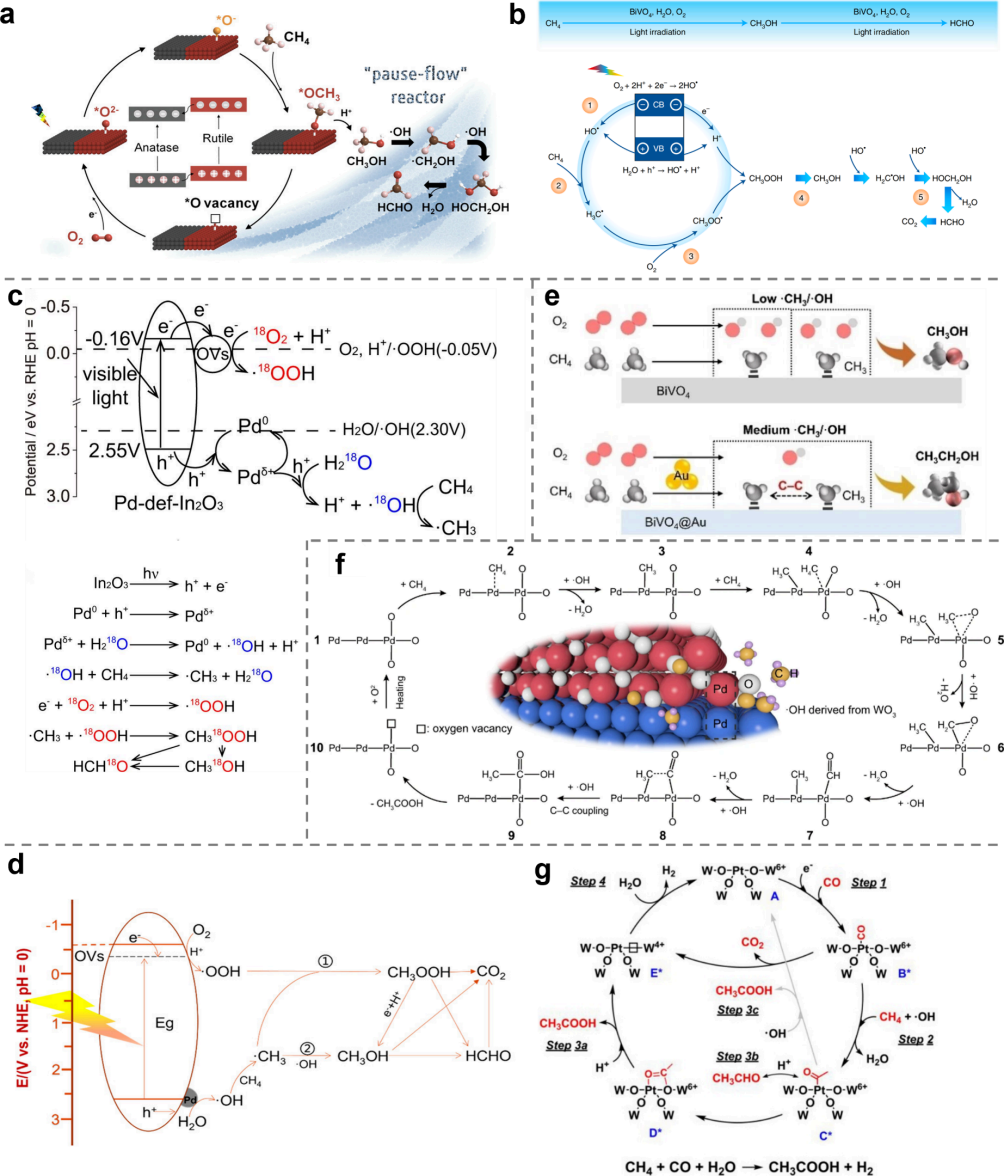
**The coupling of active species.** (**a**) Proposed mechanism of photocatalytic CH_4_ oxidation on Anatase/Rutile-TiO_2_.^86^ Adapted from ref (^86^). Copyright 2019, American Chemical Society. (**b**) Proposed photocatalytic CH_4_ oxidation mechanism over quantum-sized BiVO_4_. CB, conduction band; VB, valence band.^36^ Adapted from ref (^36^). Copyright 2021, Fan, Y. (**c**) Illustration of aerobic photocatalytic CH_4_ conversion over Pd-def-In_2_O_3_.^39^ Adapted from ref (^39^). Copyright 2022, Luo, L. (**d**) Proposed reaction mechanism over Pd-def-TiO_2_.^41^ Adapted from ref (^41^). Copyright 2022, by Wiley-VCH. (**e**) On bare BiVO_4_, the interfacial ·CH_3_/·OH ratio is low, and the photocatalytic CH_4_ conversion product is mainly CH_3_OH with O_2_ as reactant. On BiVO_4_@Au, the interfacial ·CH_3_/·OH ratio is medium, which promotes the selectivity of ethanol.^31^ Adapted with permission from ref (^31^). Copyright 2024 John Wiley and Sons. (**f**) Schematic illustration for photochemical conversion of CH_4_ to CH_3_COOH over Pd/PdO heterointerface in the presence of ·OH radicals. The numbers represent the reaction steps.^30^ Adapted with permission from ref (^30^). Copyright 2023 Zhang, W. (**g**) Scheme of the generation of photoexcited charge carriers and proposed mechanism of the photocatalytic CH_3_COOH generation process over a 4-fold oxygen coordinated Pt_1_ site. Note that from states B to E, the * marks the reduction state of NPW due to accepting photoexcited electrons from TiO_2_.^87^ Adapted with permission from ref (^87^). Copyright 2023 American Chemical Society.

In this Outlook, we emphasize the importance of jointly regulating active-species formation, species coupling, and intermediate transformation to guide overall CH_4_-conversion pathways.

C–O coupling is particularly prevalent in solution-phase photocatalytic systems, where multiple oxygen sources such as O_2_ and H_2_O coexist. Isotopic labeling experiments have been instrumental in clarifying the origin of oxygen atoms in the products. For example, for Pd single atom loaded defective In_2_O_3_ (Pd-def-In_2_O_3_) photocatalysts strong CH_3_^18^OH signals were detected in the presence of ^18^O_2_ and H_2_^16^O, while reactions performed with ^16^O_2_ and H_2_^18^O produced only CH_3_^16^OH and H_2_^18^O. These results indicated that ·OOH derived from O_2_ reduction preferably coupled with ·CH_3_ to form CH_3_OOH and subsequently CH_3_OH, whereas ·OH generated from H_2_O oxidation, mainly participated in CH_4_ activation (Figure 5c).^39^ In contrast, for photocatalysts consisting of Pd nanoparticles loaded on defective TiO_2_ (Pd-def-TiO_2_) CH_3_^18^OH has been produced irrespectively of the isotope used (H_2_^16^O + ^18^O_2_ and H_2_^18^O + ^16^O_2_).^41^ Therefore, both ·OOH from O_2_ and ·OH from H_2_O are likely to contribute to CH_3_OH formation by coupling with ·CH_3_. The initially formed CH_3_OOH and CH_3_OH then underwent further oxidation to HCHO, as illustrated in Figure 5d.

### 4.3 Comprehensive regulation of C–C and C–O formation

The interplay between C–C and C–O coupling governs the selective formation of multicarbon oxygenates, particularly C_2_H_5_OH and CH_3_COOH, the two most representative products. This section focuses on these pathways and the strategies for regulating C–C and C–O bond formation.

#### 4.3.1 C_2_H_5_OH Formation

C_2_H_5_OH formation involves C–O coupling between CH_3_ and OH species to generate ·CH_2_OH radicals, which subsequently couple with ·CH_3_ radicals to form C_2_ products. Thus, controlling the relative concentrations of oxidative species is essential. Excess of strong oxidants such as ·OH drives overoxidation to HCHO or HCOOH, whereas an insufficient oxidative strength suppresses ·CH_2_OH formation and limits C–C coupling toward C_2_H_5_OH. Previously anaerobic CH_4_ oxidation systems were used primarily due to milder oxidation capacity. For example, Zhou et al.^19^ employed Cu–PCN with a tailored band structure to regulate ·OH generation through controlled H_2_O_2_ formation from H_2_O oxidation that was decomposed *in situ* on Cu sites. This design effectively prevented excessive ·OH accumulation, thereby facilitating a stepwise CH_4_ → CH_3_OH → C_2_H_5_OH conversion pathway. Subsequent studies further emphasize the importance of maintaining a balanced ·CH_3_/·OH ratio. For instance, a three-phase Fe­(III)-cross-linked macroporous alginate hydrogel film encapsulated with a C_3_N_4_ (Fe­(III)@ACN)^88^ system enabled *in situ* H_2_O_2_ generation via H_2_O oxidation on C_3_N_4_, followed by Fenton-type ·OH production through photoinduced Fe­(II). Efficient CH_4_ mass transfer at the gas–liquid–solid interface facilitated ·CH_3_ formation, maintaining comparable ·CH_3_ and ·OH concentrations. This balance promoted partial dehydrogenation of CH_3_OH to ·CH_2_OH, which coupled with ·CH_3_ to yield C_2_H_5_OH with up to 90% selectivity among the alcohols produced. Despite the success in achieving high C_2_H_5_OH selectivity, the overall CH_4_ conversion rate remains restricted by thermodynamic boundaries. Aerobic systems were for example investigated by Zhang et al.^31^ using BiVO_4_ for C_2_H_5_OH formation (Figure 5e). They found that bare BiVO_4_ generated an excess of ·OH radicals, resulting in a low ·CH_3_/·OH ratio and predominant CH_3_OH production. After surface functionalization with Au nanoparticles, both the *in situ* generation of ·OH were optimized. This regulation resulted in a moderate ·CH_3_/·OH ratio which favored C–C coupling. As a result, the BiVO_4_@Au catalyst achieved an C_2_H_5_OH yield of 680 μmol·g^–1^·h^–1^ with a selectivity of 86%.

#### 4.3.2 CH_3_COOH Formation

CH_3_COOH formation primarily occurs via the coupling of *CH_3_ radicals with *CO species. Most mechanistic studies suggest that neutral intermediates such as CH_3_OH or CH_3_CH_2_OH are bypassed. Instead, the reaction proceeds through the nucleophilic attack of *CH_3_ on adsorbed *CO to form an acetyl intermediate (*COCH_3_), which is then oxidized and protonated to produce CH_3_COOH. The selectivity of this process strongly depends on the coexistence and spatial proximity of *CH_3_ and *CO species, as well as the catalyst’s ability to stabilize the acetyl intermediate while preventing overoxidation to CO_2_. A critical regulation strategy for steering this pathway is heterointerface engineering, which enables the concurrent generation and stabilization of *CH_3_ and *CO. Zhang et al.^30^ constructed a PdO/Pd–WO_3_ heterostructure, in which PdO provided lattice oxygen that directly oxidizes CH_4_ to generate surface *CO, while adjacent metallic Pd atoms acted as anchoring sites to stabilize *CH_3_ radicals. The intimate Pd–PdO interface facilitated efficient *CH_3_–*CO coupling, leading to the direct synthesis of CH_3_COOH from CH_4_ and H_2_O with 91.6% selectivity (Figure 5f). This example highlights that engineering redox-active oxide domains in close contact with metallic sites can synchronize the generation of distinct reactive intermediates, thereby promoting selective C–C and C–O bond formation. An alternative approach is to introduce CO as a coreactant, which circumvents the need to generate *CO exclusively from CH_4_ and thus alleviates the risk of overoxidation. This has been shown for TiO_2_-based photocatalyst modified with subnanometer ammonium phosphotungstic polyoxometalate (NPW) clusters that host isolated Pt single atoms (Pt_1_).^87^ In this system, H_2_O-derived ·OH radicals activated CH_4_ to form ·CH_3_, while the Pt_1_ centers bound and activated CO. The two intermediates then coupled to generate an *COCH_3_ species, which is further oxidized to CH_3_COOH with over 90% selectivity under ambient conditions (Figure 5g). Similarly, Li et al.^89^ reported a MoS_2_-supported Rh–Zn dual-atom catalyst integrated with TiO_2_, where Zn–OH moieties efficiently activated CH_4_ to yield *CH_3_, and Rh sites selectively adsorbed CO. The confined Rh–Zn pairs enforced spatial vicinity of the two intermediates, enabling their coupling into *COCH_3_ with a remarkably high CH_3_COOH selectivity of 96.5%.

In comparison, systems that derive *CO intrinsically from CH_4_ oxidation avoid the use of additives but are challenged by lower reactivity.^30^ In contrast, cofeeding CO ensures a sufficient surface coverage of carbonyl intermediates and therefore higher CH_3_COOH selectivity, although at the expense of using multiple carbon sources.^22^ Understanding and comparing these two strategies provides important guidance for future catalyst design: maximizing *CO availability while preventing overoxidation remains essential to realize both high activity and selectivity in photocatalytic CH_3_COOH synthesis.

### 4.4 C–X Bond Formation

When halide salts are introduced into the photocatalytic system, the generated halogen radicals can couple with ·CH_3_ to form methyl halides (CH_3_X, X = Cl, Br), which serve as valuable intermediates for CH_3_OH synthesis and pharmaceutical production. For instance, Li et al.^33^ demonstrated the photocatalytic conversion of CH_4_ to CH_3_Cl over Ag_2_O/NaTaO_3_ using NaCl solutions. CH_4_ were initially activated by Ag_2_O to generate ·CH_3_. Upon light irradiation, photogenerated holes oxidized Cl^–^ to form ·Cl, which subsequently coupled with ·CH_3_ to yield CH_3_Cl, while electrons reduced H^+^ into H_2_. In their system, Ag_2_O effectively suppressed ·OH formation and stabilized absorbed ·Cl, as shown by electron paramagnetic resonance spectroscopy.^33^ Similarly, Ma et al.^32^ employed sodium halides (NaCl, NaBr) as halogen sources, achieving a high selectivity (86.7%) for CH_3_X formation in a Cu-TiO_2_ system. Importantly the utilization of seawater was also shown to be feasible.^32^ Evidently the selective CH_3_X formation requires balancing of several competing processes, namely, the activation of CH_4_ to ·CH_3_, the oxidation of X^–^ to ·X, and the suppression of undesired ·CH_3_ oxidation by reactive oxygen species.

## 5 The Conversion of the Intermediate Products

The intermediate products discussed here are thermodynamically stable neutral C_1_–C_2_ species, mainly C_2_H_6_, CH_3_OH, and CH_3_OOH. In some photocatalytic systems, these compounds serve as final products, while in others, they act as precursors for the generation of among others C_2_H_4_, HCHO, HCOOH or C_2_H_5_OH (Figure 6a). Their fate, whether they remain stable or undergo subsequent transformation, depends strongly on the catalyst design and reaction environment. In addition to thermodynamic factors, kinetic effects such as intermediate residence time and mass transport play important roles in determining selectivity. Rapid desorption or transport of intermediates can suppress further conversion, whereas prolonged surface residence increases the likelihood of sequential oxidation or coupling reactions. Therefore, controlling both the catalytic sites and reaction conditions that regulate intermediate lifetime is essential for directing product selectivity in photocatalytic CH_4_ conversion.

**Figure 6:**
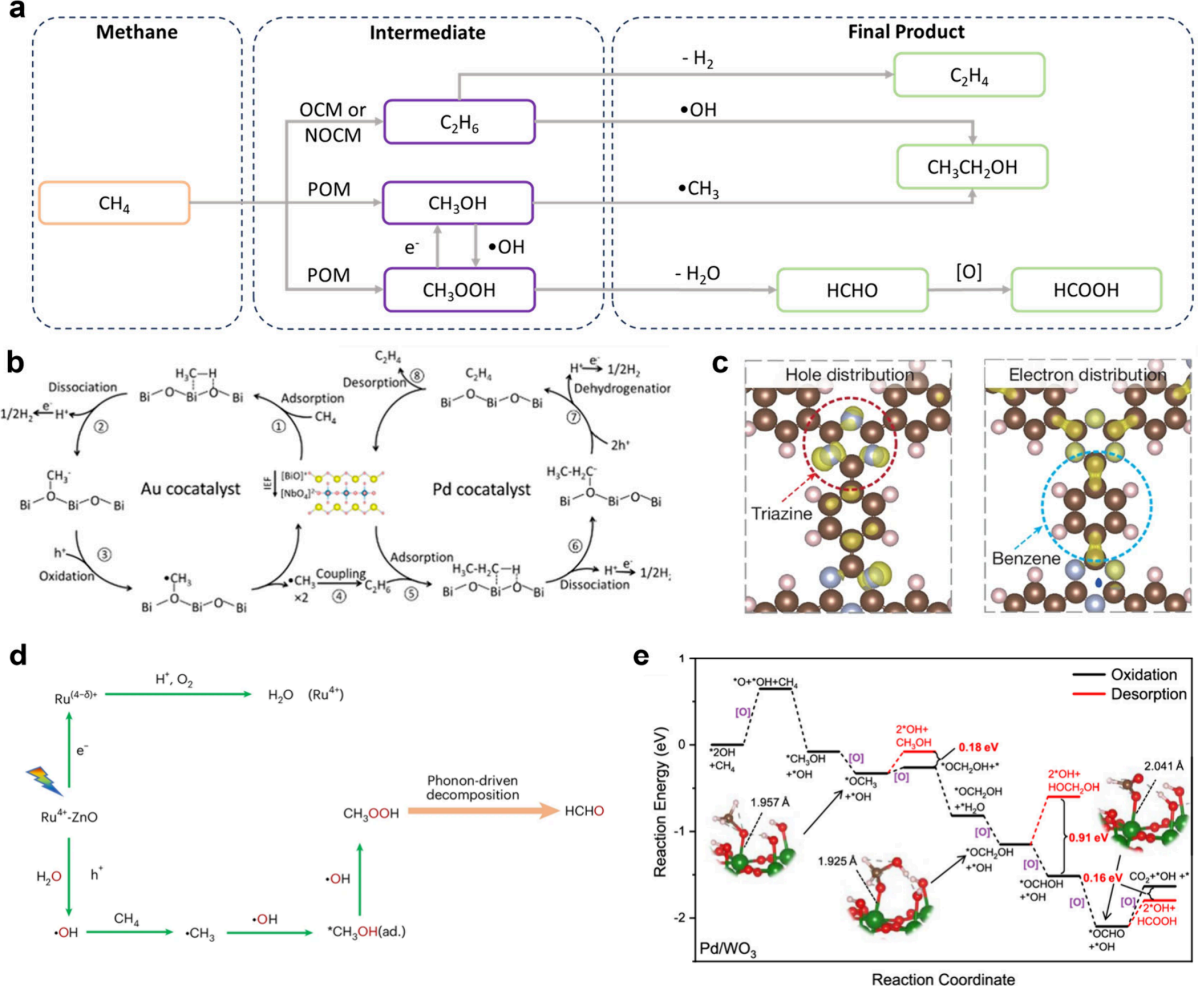
**The conversion of intermediate product.** (**a**) Scheme of CH_4_ to intermediate product and then to final product. (**b**) Schematic illustration for photocatalytic conversion of CH_4_ to C_2_H_4_ over the optimized Au–Pd/BNOF.^38^ Adapted with permission from ref (^38^). Copyright 2023 American Chemical Society. (**c**) Spatial distribution of the HOMO (left) and LUMO (right) of CTF-1 catalyst, which reflect the regions that preferentially host photogenerated holes and electrons after excitation.^91^ Adapted with permission from ref (^91^). Copyright 2025 Springer Nature. (**d**) The proposed reaction pathways of photon–phonon-driven cascade catalysis for CH_4_ oxidation to HCHO. ad., adsorbed.^92^ Adapted with permission from ref (^92^). Copyright 2024 Springer Nature. (**e**) Proposed methane oxidation pathway over Pd/WO_3_, where [O] is the active species from O_2_ activation on Pd.^93^ Adapted with permission from ref (^93^). Copyright 2024 American Chemical Society.

For C_2_H_6_, the main transformation pathway is dehydrogenation to C_2_H_4_. A powerful materials-based strategy to achieve this involves designing tandem systems in which coupling and dehydrogenation sites are spatially integrated. For example, Tang et al. constructed a tandem Au–Pd/Bi_2_NbO_3_F catalyst for photocatalytic nonoxidative CH_4_ coupling, where Au sites promoted ·CH_3_ coupling to form C_2_H_6_, and adjacent Pd sites catalyzed its subsequent dehydrogenation to C_2_H_4_, achieving 63% selectivity (Figure 6b).^38^ A similar concept was demonstrated in an Ag–Pd/ZnO system, which achieved 50% selectivity for C_2_H_4_.^90^ In this system, photogenerated holes enriched at ZnO lattice oxygen sites activated CH_4_ and facilitated C–H bond cleavage resulting in *CH_3_ formation. The *CH_3_ species were observed to be coupled on Pd single atoms, yielding C_2_H_6_. This was then found to deprotonate to C_2_H_5_ and migrate to Ag sites, where further dehydrogenation produced C_2_H_4_ as the final product. C_2_H_6_ can also be selectively converted to C_2_H_5_OH through controlled oxidation. Similar to the dehydrogenation process, this mechanism benefits from spatial separation of redox sites, as demonstrated for a covalent triazine framework (CTF-1) photocatalyst featuring an intramolecular heterojunction (Figure 6c).^91^ In this system, photogenerated electrons accumulated on the carbon sites of the benzene motifs, while holes localized on the nitrogen sites of the triazine units. The benzene motifs catalyzed H_2_O oxidation to generate ·OH radicals, which activated CH_4_ to form ·CH_3_ radicals that coupled to yield the C_2_H_6_ intermediate. Meanwhile, the spatially separated triazine motifs produced controlled oxidants that selectively oxidize C_2_H_6_ with surface-adsorbed O_2_, achieving 78.6% selectivity toward C_2_H_5_OH in a packed-bed flow reactor.

As an unstable and reactive intermediate, CH_3_OOH can be further transformed into either HCHO or CH_3_OH under specific conditions. It is known that CH_3_OOH gradually decomposed into HCHO at room temperature.^40^ The conversion of CH_3_OOH to HCHO is generally promoted by thermal activation or radical-mediated processes. For instance, Xu et al. demonstrated a photothermal process achieving up to 90% selectivity toward HCHO over Ru single atoms supported on ZnO at 150 °C.^92^ In their system, photocatalytic CH_4_ oxidation initially produced CH_3_OOH, which subsequently underwent selective thermal decomposition to yield HCHO and H_2_O (Figure 6d). These findings indicate that mild heating or radical-rich environments can effectively steer CH_3_OOH oxidation toward HCHO. Conversely, under reductive conditions with abundant electrons or hydrogen donors, CH_3_OOH can be hydrogenated to CH_3_OH via a proton-coupled electron transfer mechanism. This reduction typically occurs on cocatalyst surfaces through photogenerated electrons, as discussed in Section 4.2.

CH_3_OH can act as an intermediate for the formation of either HCHO or C_2_H_5_OH. The conversion pathway to HCHO has been discussed previously; here, we focus on C_2_H_5_OH formation, in which CH_3_OH serves as a key intermediate. In this pathway, CH_3_OH undergoes partial dehydrogenation to generate ·CH_2_OH radicals, which subsequently couple with ·CH_3_ radicals to form CH_3_CH_2_OH. Zhou et al. confirmed this mechanism through a series of control experiments using a Cu-PCN photocatalyst.^19^ When additional CH_3_OH was introduced into the photocatalytic system, the yield of C_2_H_5_OH increased significantly for both pristine PCN and Cu–PCN photocatalysts, highlighting the role of CH_3_OH as a reactive intermediate. Furthermore, experiments conducted with CH_3_OH alone (in the absence of CH_4_) on Cu–PCN yielded less C_2_H_5_OH than those performed with CH_4_ present, likely due to the limited availability of ·CH_3_ radicals. Collectively, these findings support a stepwise CH_4_ → CH_3_OH → C_2_H_5_OH conversion pathway.

HCHO, as well as its hydrated form HOCH_2_OH, can be further oxidized to HCOOH. This transformation typically proceeds through a two-electron oxidation process rather than bond cleavage. Electrophilic surface oxygen species play a crucial role by serving as hydrogen acceptors from the O–H bond to form *OCHO intermediates. Jiang et al.^93^ demonstrated this mechanism using a Pd/WO_3_ catalyst (Figure 6e), where Pd nanoparticles generated electrophilic *O species from O_2_. In combination with an optimal adsorption energy for *OCHO provided by WO_3_, HCOOH was obtained with a 62% selectivity. Following this work, Zhai et al.^42^ reported a higher selectivity of 84% using Pt nanoparticle-decorated WO_3_ in H_2_SO_4_ solution. In their system, Pt nanoparticles can facilitate electron transfer and H_2_SO_4_ provided sufficient protons to promote O_2_ activation via a proton-coupled electron transfer process to generate abundant ·OH radicals, while simultaneously enhancing *CH_2_O adsorption and suppressing premature HCHO desorption.

## 6 Perspective

Photocatalytic CH_4_ conversion enables the selective formation of oxygenates and hydrocarbons through CH_4_ partial oxidation and coupling reactions, offering both economic and environmental advantages. Unlike conventional photocatalytic reactions that involve relatively simple steps, photocatalytic CH_4_ conversion comprises multiple elementary processes and involves a variety of reactive species. As a result, its overall efficiency, product diversity, and mechanistic understanding remain limited, largely due to the absence of a comprehensive framework for describing the CH_4_ oxidation process. In this outlook, photocatalytic CH_4_ conversion is organized into three fundamental steps: (1) formation of reactive species, (2) coupling of reactive species, and (3) transformation of intermediate products. We summarize representative reaction pathways within each step and present and discuss strategies for regulating reaction activity and selectivity that have been recently reported (Figure 7). These strategies include controlling the nature and surface interactions of active species through electronic structure design based on d-band center theory or directing intermediate conversion via catalyst engineering. This analysis indicates that the strategic regulation of each step has facilitated substantial progress in the optimization of photocatalytic CH_4_ conversion. Nevertheless, the diversity of products remains limited, and the majority of current studies focus on tuning individual steps in isolation. Therefore, an integrated strategy accounting for the interplay among multiple reaction steps is proposed as being essential for advancing efficient and selective CH_4_ conversion.

**Figure 7:**
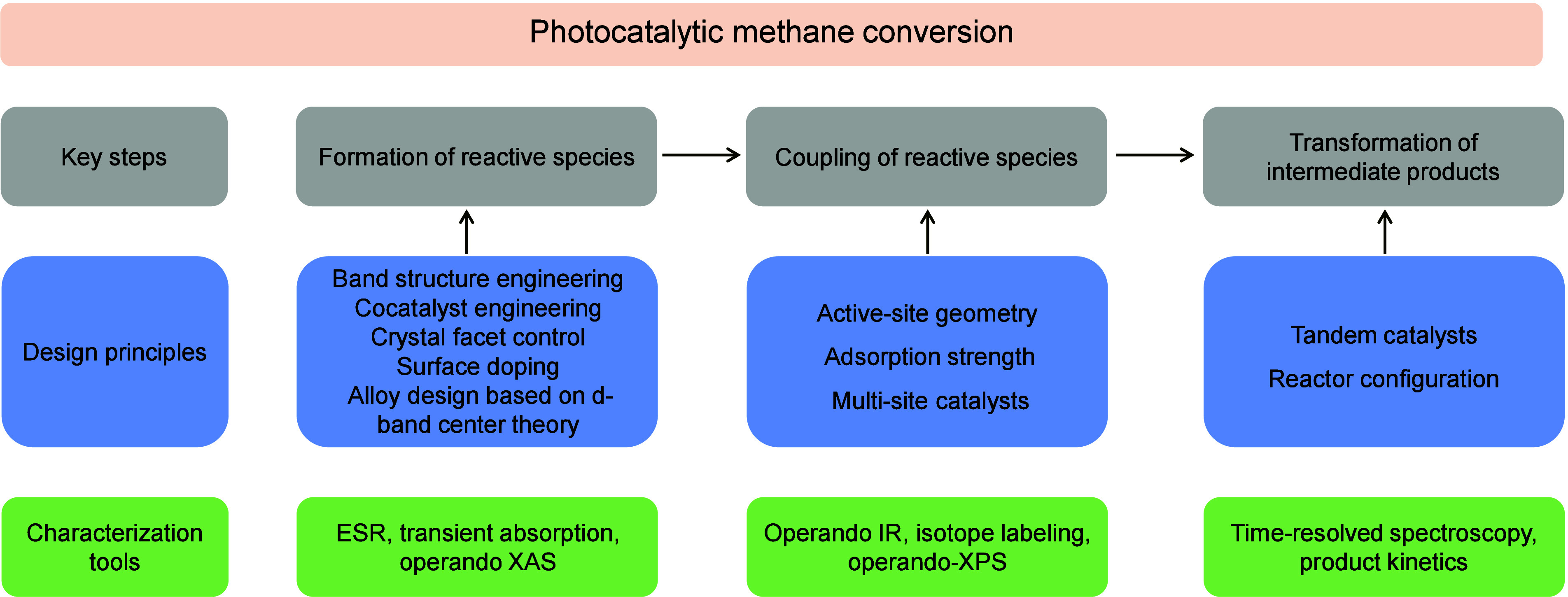
Roadmap for photocatalytic methane conversion: linking mechanistic steps, catalyst design, and characterization tools.

From the perspective of active-species formation, the major reactive oxygen and carbon species involved in photocatalytic CH_4_ conversion, such as ·OH, ·OOH, O_L_^–^, M–O, ·CH_3_, CH_2_ species and *CO, are now broadly recognized. To control the formation of active species, several design strategies can be considered (Figure 7). For example, tuning the size and coordination structure of cocatalysts,^94^ as well as the band structure and crystal facets of metal oxides, can regulate the selective formation of oxygen reactive species. Surface doping can be used to modulate the activity of lattice oxygen (O_L_^–^). In addition, shifting the d-band position of cocatalyst metals through alloying can help regulate the adsorption and reactivity of carbon-containing intermediates. However, their dynamic states during reaction, including concentration, adsorption configuration, and surface lifetime, remain poorly understood, despite their decisive impact on reaction efficiency and selectivity. Elucidating these dynamics requires advanced *in situ* and *operando* characterization. *In situ* ESR spectroscopy enables direct detection of short-lived radicals (e.g., ·OH, ·OOH, ·CH_3_), providing information on radical flux and lifetime under illumination. *Operando* infrared spectroscopy (DRIFTS or ATR-FTIR), especially when combined with isotopic labeling, identifies surface-bound carbon and oxygen intermediates and tracks their evolution. *Operando* X-ray absorption spectroscopy probes transient metal oxidation states and coordination environments associated with M–O formation and lattice oxygen activation, while near-ambient pressure XPS monitors surface oxygen species, adsorbates and potentially also vacancies under more realistic conditions. Time-resolved optical spectroscopies have been shown to correlate charge-carrier dynamics with reactive-species generation. In future, efforts should therefore move beyond static identification to quantitative measurement of species lifetimes, concentration ratios, and spatial localization, and their correlation with product selectivity and catalyst stability. For instance, radical lifetimes and steady-state concentrations measured by *in situ ESR* indicate whether CH_4_ activation or downstream coupling limits the pathway. *Operando* IR spectroscopy can track surface coverage and adsorption configurations to identify bottlenecks in C–C or C–O bond formation, while *operando* XPS and site-sensitive spectroscopies provide insight into the spatial localization of reactive species and the roles of different catalytic sites. In addition, time-resolved spectroscopies linking charge-carrier dynamics with reactive-species generation help determine whether photophysics or surface chemistry governs overall efficiency. Together, these quantitative observables transform operando techniques from descriptive tools into mechanistic diagnostics for identifying the steps controlling reaction pathways and selectivity.

Only through close collaboration across semiconductor physics, catalysis chemistry, and chemical engineering can photocatalytic CH_4_ conversion be advanced toward practical implementation.

Although several strategies have been developed to promote the formation of specific species, multiple reactive species typically coexist and operate simultaneously. Achieving precise control over a target species while suppressing undesired ones, particularly to balance CH_4_ activation against overoxidation, remains an unsolved challenge. Oxygen active species undeniably dominate CH_4_ activation but also readily promote deep oxidation. The governing rules that determine whether selective activation proceeds via nucleophilic or electrophilic oxygen attack, as well as the associated reaction kinetics, remain poorly understood. The strong tendency toward overoxidation therefore motivates exploration of alternative activation motifs, such as sulfur- or halogen-centered species, which may enable fundamentally different reaction pathways and expand the accessible product space.^27^ Reactive carbon species are short-lived and difficult to stabilize, consistent with the limited mechanistic studies available. Progress in the field will particularly require to develop more effective capture methods of carbon-intermediates and higher-resolution spectroscopies, along with the design of catalysts capable of stabilizing carbon species, such as Cu-based or carbon-support materials.

From the perspective of coupling of active species, current research is still limited, with most studies focusing on identifying species types and tuning their concentrations. However, efficient coupling depends not only on concentration but also, as outlined in Section 5 on the spatial arrangement of reactive species. While the control of species distribution is fundamental, the regulation of their relative positions offers an additional lever for the precise control of coupling pathways, especially for multicarbon products. Designing dual-site or multisite catalysts capable of positioning distinct species in proximity may significantly enhance both C–C and C–O coupling as has been demonstrated already in same cases.^21,89,95^ Considering these two coupling modes simultaneously appears to be most essential for steering product selectivity.

From the perspective of intermediate-product transformation, photocatalytic synthesis of multicarbon and structurally complex products from CH_4_ represents an attractive but underexplored direction. Although several active intermediate products have been identified, deliberate catalyst or system designs that convert these intermediates through tandem pathways remain scarce.^38,91^ Achieving such transformations requires highly integrated catalyst architectures together with advanced reaction-system engineering. In particular, reactor parameters such as residence time, oxidant distribution, and phase contact can strongly influence the fate of intermediates and determine whether they undergo further coupling or undesired overoxidation. Reactor configurations that spatially or temporally separate CH_4_ activation from downstream oxidation steps may therefore help preserve reactive intermediates and improve selectivity toward C_2+_ products. In this sense, the selective transformation of intermediates is not only a mechanistic step but also a system-level challenge that reflects the level of precision achievable in pathway control and reactor design.

Despite substantial progress, photocatalytic CH_4_ conversion still faces challenges related to low efficiency, limited product diversity, and poor scalability of reaction systems. In this Outlook, we emphasize the importance of jointly regulating active-species formation, species coupling, and intermediate transformation to guide overall CH_4_-conversion pathways. Furthermore, careful design of semiconductor band structures and control over the photoexcitation process are equally crucial. Only through close collaboration across semiconductor physics, catalysis chemistry, and chemical engineering can photocatalytic CH_4_ conversion be advanced toward practical implementation.
